# *Schisandrin* protects against ulcerative colitis by inhibiting the SGK1/NLRP3 signaling pathway and reshaping gut microbiota in mice

**DOI:** 10.1186/s13020-023-00815-8

**Published:** 2023-09-06

**Authors:** Xiaohu Wang, Chaozhuang Shen, Xingwen Wang, Jin Tang, Zijing Wu, Yunzhe Huang, Wenxin Shao, Kuo Geng, Haitang Xie, Zhichen Pu

**Affiliations:** 1 Anhui Provincial Center for Drug Clinical Evaluation, Yijishan Hospital of Wannan Medical College, No. 2, Zheshan West Road, Jinghu District, Wuhu, 241000 China; 2 Graduate School of Wannan Medical College, No.22, Wenchang West Road, Yijiang District, Wuhu, 241000 China; 3 Department of Pharmacy, Bengbu First People’s Hospital, Bengbu, 233000 China

**Keywords:** *Schisandra chinensis* (Turcz.) Baill., *Schisandrin*, Ulcerative colitis, NLRP3, Gut microbiota

## Abstract

**Background:**

According to the *Chinese Pharmacopoeia*, the fruit of *Schisandra chinensis (Turcz.)* Baill. (SC) is an important traditional Chinese medicine that can be used to treat diarrhea. Despite the increasing research on the anti-inflammatory and anti-oxidant aspects of SC, the studies on the anti-ulcerative colitis of Schisandrin (SCH), the main constituent of SC, are relatively few.

**Methods:**

The mice used in the study were randomly distributed into 6 groups: control, model, 5-ASA, and SCH (20, 40, 80 mg/kg/d). The mice in the model group were administered 3% (w/v) dextran sulfate sodium (DSS) through drinking water for 7 days, and the various parameters of disease activity index (DAI) such as body weight loss, stool consistency, and gross blood were measured. ELISA was used to detect inflammatory factors, and bioinformatics combined with transcriptome analysis was done to screen and verify relevant targets. 16S rDNA high-throughput sequencing was used to analyze the composition of the gut microbiota(GM), while mass spectrometry was done to analyze the changes in the content of bile acids (BAs) in the intestine.

**Results:**

Mice treated with SCH experienced significant weight gain, effectively alleviating the severity of colitis, and decreasing the levels of inflammatory factors such as TNF-α, IL-1β, IL-18, IL-6, and other related proteins (NLRP3, Caspase-1, SGK1) in UC mice. Furthermore, the analysis of GM and BAs in mice revealed that SCH increased the relative abundance of *Lactobacilli spp*, reduced the relative abundance of *Bacteroides*, and promoted the conversion of primary BAs to secondary BAs. These effects contributed to a significant improvement in the DSS-induced GM imbalance and the maintenance of intestinal homeostasis.

**Conclusion:**

It seems that there is a close relationship between the SCH mechanism and the regulation of SGK1/NLRP3 pathway and the restoration of GM balance. Therefore, it can be concluded that SCH could be a potential drug for the treatment of UC.

## Background

Ulcerative colitis (UC), a type of chronic non-specific inflammation with an unknown etiology, is generally believed that the pathogenesis of UC could be caused by immune system disorders, genetics, environment and microbial infections and other factors [1].

Despite surgical interventions, many UC patients still face unmet treatment needs, leading to a significant financial burden on their families. Moreover, UC not only negatively impacts the quality of life for patients but also increases the risk of colon cancer. As a result, the World Health Organization has classified UC as a modern refractory disease [2]. Currently, the primary treatment approaches for UC revolve around anti-inflammatory and immunosuppressive strategies.

Inflammation is the body's self-protective response during pathogenesis, but it can sometimes lead to the development of inflammatory disease and cause damage to the body. Studies have shown that the occurrence and development of UC are closely related to the NLRP3 inflammasome [3, 4]. The NLRP3 inflammasome activate pro-caspase-1 downstream and cleave it into activated caspase-1, which acts on pro-IL-1β and pro-IL-18, to finally form mature IL-1β and IL-18. Hence, the reduction of IL-1 β and IL-18 are important mechanisms for the prevention and treatment of UC [5].

Similarly, GM (Gut Microbiota) also plays significant roles in the UC occurrence and development. GM hosts and participates were symbiotic in vital metabolic, immune, and intestinal protective effects in vivo. When the species, numbers and ratios of normal flora turn abnormal, microbial ecology become dysregulated, the immune system imbalanced and metabolism disturbed, leading to intestinal mucosal damage [6]. Consequently, restoring the balance of GM through drug interventions becomes an essential approach in the treatment of UC [7].

The fruit of *Schisandra chinensis* (Turcz.) Baill. (SC) is an important herbal medicine which has been widely used in Traditional Chinese Medicine (TCM) for the treatment of intestinal disorders [8, 9]. SC is a TCM documented in the Chinese Pharmacopoeia (2020) for its liver protective and diarrhea treatment properties. Schisandrin (SCH), one of the main biologically active lignan components, is primarily derived from the peel and mature seed coat of SC.

SCH possesses potent pharmacological properties and has been found to exhibit beneficial effects in the treatment of various diseases. These effects include anti-inflammatory and antioxidant properties [10, 11], antidepressant effects [12], cerebral neurocytoprotection and mitochondrial function protection [13].

Moreover, SCH is known to significantly inhibit the activation of the NLRP3 inflammasome during acute lung injury, reducing apoptosis and also declined the levels of IL-1β, IL-18, and IL-6 [14]. However, it is unclear whether SCH possesses an inhibitory effect on NLRP3 inflammasome in the case of UC [9]. Other lignans in SC have anti-colitis effects by improving the GM balance and reducing oxidative stress [15, 16]. SCH is the main component in SC [17], and there seemed rarely researches on its anti-colitis properties [18]. In a situation where no specific medicine is available for UC clinical treatment today, it could be of great value to fully explore active ingredients in SC for their potential effects in UC treatment.

To verify this conjecture, we examined the protective effects of SCH on UC mice and explored the underlying mechanism, for the first time, in light of GM composition and NLRP3 inflammasome. Our results provide a theoretical and experimental basis for SCH against UC.

## Methods and methods

### Drugs and reagents

SCH was obtained from Sichuan CSITE Bio-technology Co., Ltd. (SiChuan, China, purity > 98%). DSS (Dextran Sulfate Sodium Salt) was purchased from APEx Biomedicals (California, USA). 5-Aminosalicylic acid (5-ASA) was purchased from Aladdin (Shanghai, China). Mouse ELISA kits for IL-6, IL-1β, IL-18, MPO, RELMβ, TFF3 and TNF-α were obtained from MultiSciences Biotech Co., Ltd. (Zhejiang, China). SGK1 and MUC-2 antibody was obtained from Abcam. The chemicals and reagents used in the pharmacokinetics are detailed in the previous research basis [17].

### Pharmacokinetic analysis of SCE in rats

All experiments involving animals were approved by the Animal Care and Use Committee of Wannan Medical College. The SPF grade SD rats (180–220 g) were purchased from Qinglongshan (Nanjing, Suzhou, China) Animal Testing Center, license number SCXK (Su) 2017-0001. Rats were given 0.95 g/kg of *Schisandra chinensis* (Turcz.) Baill. Extract (SCE) by gavage. 0.25 mL of blood were collected separately at 0 h, 0.25 h, 0.5 h, 1 h, 2 h, 4 h, 6 h, 8 h, 12 h, 24 h, and 36 h after administration from the orbital vein of rats. Blood concentration were then measured applying HPLC–MS/MS established by the research group [17]. PK parameters of seven lignans were calculated by Phoenix WinNonlin 6.0. Chromatographic separation was performed on an Agilent ZORBAX Eclipse XDB-C18 (4.6 mm × 150 mm, 5 μm) column, the mobile phase was 90% methanol (containing 0.1% formic acid) and water (containing 0.1% formic acid, 5 mmol ammonium acetate), and the flow rate was 0.5 mL/min, the column temperature was 30 °C, and the injection volume was 10 μL. Electrospray positive ion multiplier reaction monitoring (MRM) was used as the detector.

### Animal experimentation

Male C57BL/6 mice (5–6 weeks, 18–20 g) were all from Qinglongshan Animal Testing Center, Suzhou City, China. The mice were randomly divided into 6 groups with 8 mice in each group, including control group, the UC model group, the 5-ASA group (50 mg/kg/d), and the SCH treatment group (20, 40, 80 mg/kg/d).

Modeling and administration methods are shown in Fig. 2A, except for the mice in the control group, the mice in each group were induced by 3.0% DSS in drinking water for 7 days, and the SCH group was administered by gavage for 3 days in advance, once a day. The body weight of mice in each group was measured daily, feces and bleeding were also recorded, and the DAI was evaluated using the method of Dong et al. [19], DAI = (weight loss [%] + presence or absence offecal blood + stool consistency)/3. weight loss (scored as: 0, none; 1, 1–5%; 2, 5–10%; 3, 10–20%; 4, over 20%), presence or absence offecal blood (scored as: 0, negative hemoccult; 2, positive hemoccult; 4, gross bleeding) and stool consistency (scored as: 0, well-formed pellets;2, loose stools; 4, diarrhea).

Next, male C57BL/6 mice (n = 24) were randomly assigned into four groups: control group, colitis group, Nigericin (Nig) + SCH (40 mg/kg/d) group, and Nig group. Nig is an antibiotic from Streptomyces hygroscopicus and is a NLRP3 activator.

Intraperitoneal administration of Nigericin to the colitis group of mice exacerbates UC. The control group and colitis group were treated the same way as above. In the Nig and SCH + Nig group, mice were induced to drink water for 7 days with 3.0% DSS, and 0.5 mg/kg of Nigericin was injected intraperitoneally every day.

After the experiment was completed, the mice were anesthetized with isoflurane (3%), peripheral blood was collected from the submandibular gland vein, and the supernatant was removed after centrifugation for future use. Then, the mice were executed by cervical dislocation under anesthesia. About 1 cm of colon was collected and fixed in 4% paraformaldehyde for histological and immunohistochemical examinations. The colonic contents and remainder of the colon were then collected and stored at −80 °C for other experiments.

All animals were fed on standard chow in a climate-controlled facility at 22 ± 2 ℃ and relative humidity 50 ± 10% with dark/light cycle of 12 h and were given free access to water ad libitum. Before the experiment, all animals were adaptively fed for 1 week. In order to prevent contamination, the animals were housed in an intelligent IVC system and the corn cob bedding (6 mesh) in the cages was changed daily.

### Colon tissue transcriptome analysis

The colons of the mice in the model group, the control group and the SCH group were emptied and then quickly frozen in a −80 °C refrigerator. Total RNA was extracted from colon tissue using TRIzol reagent (Invitrogen, Thermo Scientific, Waltham, Massachusetts, USA), and mRNA sequencing was performed by Majorbio Corporation (Shanghai, China) [20]. mRNA sequencing was based on the Illumina Novaseq 6000 sequencing platform. Nano drop 2000 was used to detect the concentration and purity of the extracted RNA, agarose gel electrophoresis was applied to detect RNA integrity, and Agilent 2100 was used to determine the RIN value. Sequencing experiments used Illumina TruseqTM RNA sample prep Kit method for library construction, only high-quality samples (OD260/280 = 1.8–2.2, OD260/230 ≥ 2.0, RIN ≥ 6.528 s:18S ≥ 1.0, > 50 μg) were included Analysis, and finally data analysis, information mining.

### Bioinformatics analysis

We downloaded the gene expression dataset numbered GSE13367 and sample clinical information from the Gene Expression Omnibus (GEO) of the National Center for Biotechnology Information (NCBI), of which the GSE13367 dataset contains 16 cases UC samples and 20 control samples were screened for differentially expressed genes (DEGs) using GEO2R [21] and R studio, and protein–protein interaction (PPI) network analysis was performed on DEGs on the STRING website. According to the analysis results of human UC transcriptome and mice UC transcriptome, we selected suitable targets for further research, and used molecular docking technology [22] for verification to predict the binding of SCH to the target. 2.5.0 software for final visualization.

### 16S rRNA sequencing

Under normal circumstances, the GM maintains a dynamic balance in the body; once the balance is disrupted, GM imbalance occurs, which is manifested by changes in the diversity, structure and bacterial abundance of the GM, especially probiotics and the increase of pathogenic bacteria are the potential pathogenesis of UC. In addition, the GM also interacts with bile acids (BAs) metabolism, immune system and intestinal mucosal barrier, and aggravates or alleviates UC through the above mechanisms.

Colon and cecal bacterial DNA from 5 mice in each group (control, model, SCH) was extracted and analyzed in accordance with previous report [23]. Briefly, the V3-V4 region of the 16S rRNA genes was amplified by PCR. The PCR products were examined by 2% agarose electrophoresis and then purified and quantified. The sequencing of purified amplicons was achieved through an Illumina MiSeq platform (Majorbio Biopharm Technology Co., Ltd., Shanghai, China) according to the standard protocols. The V3-V4 region of the 16S rRNA genes was amplified with primers 338F (5′-ACTCCTACGGGAGGCAGCAG-3′) and 806R (5′-GGACTACHVGGGTWTCTAAT-3′).

### Determination of BAs content

Weigh 100 mg of the contents of the cecum, add 1 mL of methanol, ultrasonically extract for 30 min, centrifuge at 10,000 r/min for 10 min, take 800 μL of the supernatant to dry with a nitrogen blower, reconstitute the residue with 80 μL of 70% methanol, and centrifuge again with the same parameters after fully dissolving. The clear was analyzed by HPLC–MS/MS. ESI-negative ion mode detection was used, with the following ion source parameters: IS (V):2.00; CUR:30; CAD: Medium; Source Temperature (℃):580; GS1:50; GS2:50. The conditions of gradient system are shown in Table 1 (mobile phase: A (water: acetonitrile, 10:1,1 mmol/L ammonium acetate); B (Acetonitrile: isopropanol, 1:1), injection volume 5 µL).

**Table 1:** Mobile phase gradient conditions

Time(min)	Flow(ml/min)	A%	B%
0.0	0.26	100	0
1	0.26	100	0
2	0.26	60	40
6	0.26	50	50
8	0.26	30	70
9	0.26	0	100
12	0.26	0	100
12.1	0.26	100	0
13	0.26	100	0

### ELISA assay

Blood was centrifuged at 1000 g at 4 °C for 10 min and serum was collected and saved at −80 °C for other experiment. Blood or cell samples were collected and used to measure TNF-α (H052-1), IL-6(H007-1-1), IL-18 (H015) and IL-1β (H002) levels using TNF-α, IL-6, IL-18 and IL-1β ELISA kits (Nanjing Jiancheng Biological Engineering Institute, Nanjing, China) following the manufacturer’s instructions.

50 mg of colon tissue were taken and cut into pieces. Nine times the volume of normal saline was then added in the tissue pieces, and tissue homogenization was conducted at last. The prepared homogenate was centrifuged at 2000 r/min for 10 min and the supernatant was collected. RELMβ and TFF3 levels were measured using RELMβ and TFF3 ELISA kits (Nanjing Jiancheng Bioengineering Institute, China) following the manufacturer’s instructions.

Serum and tissue (colon, liver) were treated in the same manner as above; 50 mg of intestinal contents were weighed and placed in a 5 mL centrifuge tube with 2 mL of methanol added. After vortex mixing, centrifuge at 4000 rpm for 5 min was performed, and the supernatant was taken. Total BAs levels were measured using an ELISA kit (Nanjing Jiancheng Bioengineering Institute, China) following the manufacturer's instructions.

### Western blotting analysis

Colon tissue samples or cell samples or supernatant samples were split in ice using RIPA assay (Beyotime). Total proteins were quantified using BCA assay (Beyotime) and were electrophoresed on 10% SDS-acrylamide gels. Western blotting was performed as previously described [24], using the following antibodies: NLRP3 (sc-66846, 1:500, Santa Cruz, USA); caspase-1 (c-1780, 1:500, Santa Cruz, USA); SGK1 (1:1000, Abcam) and β-actin (BS6007MH, 1:5000, Bioworld Technology, Inc.)

### H&E, Periodic acid Schiff and Alcian Blue examination (AB-PAS)

Colon tissue samples were acquired and fixed with 4% paraformaldehyde for 24 h. Colon tissues were then dehydrated, embedded in paraffin, and sliced to 5 µm thickness sections. Sections were stained with hematoxylin and eosin (H&E), PAS and Alcian Blue for 5 min. colon tissue samples were observed using an inverted fluorescence microscope (Zeiss Axio Observer A1; Carl Zeiss AG, Oberkochen, Germany).

### Immunohistochemistry

Colon tissue paraffin sections were taken, dewaxed, and rehydrated with ethanol gradient; 40 mL of pH 8.0 EDTA repair solution was added to heat antigen retrieval; PBS were rinsed for 3 times, primary antibody was add, incubate was performed afterwards at 37 °C for 60 min. PBS was rinsed again for 3 times, DAB chromogenic reagent was added, color development was stopped, and then back-stain, wash with water, dehydrate, clear, and mount.

### Immunofluorescence

Colon tissue samples were fixed in 4% paraformaldehyde, paraffin-embedded and with TBST for 3 times, sections were incubated with secondary peroxidase-conjugated goat anti-rabbit IgG (1:100, Santa Cruz Biotechnology) antibody for 2 h at room temperature. After washing with TBST for three times, sections were stained with DAPI for 15 min at darkness. Colon tissue samples were observed using fluorescence microscope (Zeiss Axio Observer A1, Germany)then sectioned into 5 μm slices. Slices samples were incubated with 0.25% Tris-X100 for 10 min at room temperature for permeabilization and repaired using citric acid for 10 min at 95 °C. Slices samples were blocked with 5% BSA in TBS for 1 h at room temperature and incubated with MUC-2 (1:1000) at 4 °C overnight.

### Cell culture and treatment

THP-1 cell was purchased from Shanghai Cell Institute Country Cell Bank and maintained in RPMI 1640 medium (Gibco) with 10% heat-inactivated fetal bovine serum (Gibco) under a humidified 5% (v/v) CO_2_ atmosphere at 37 °C. THP-1 cell was induced with 100 nM of PMA for 24 h, and treated with 100 ng/mL of LPS for 4 h and then pulsed with 1 mM of ATP for 30 min, or treated with 20/40/80 μM of SCH, 100 ng/mL of LPS for 4 h and and then for 2 mM of ATP 30 min as previously described protocols [25].

### Statistical analysis

Data were expressed as mean ± SEM. Multiple comparisons were used GraphPad Prism 8 to perform by one-way ANOVA followed by Tukey’s post-test or Kruskal–Wallis test followed by Dunn’s post hoc test. *p* < 0.05 were considered statistically significant.

## Results

### Pharmacokinetic analysis of SCE in rats

**Fig. 1:**
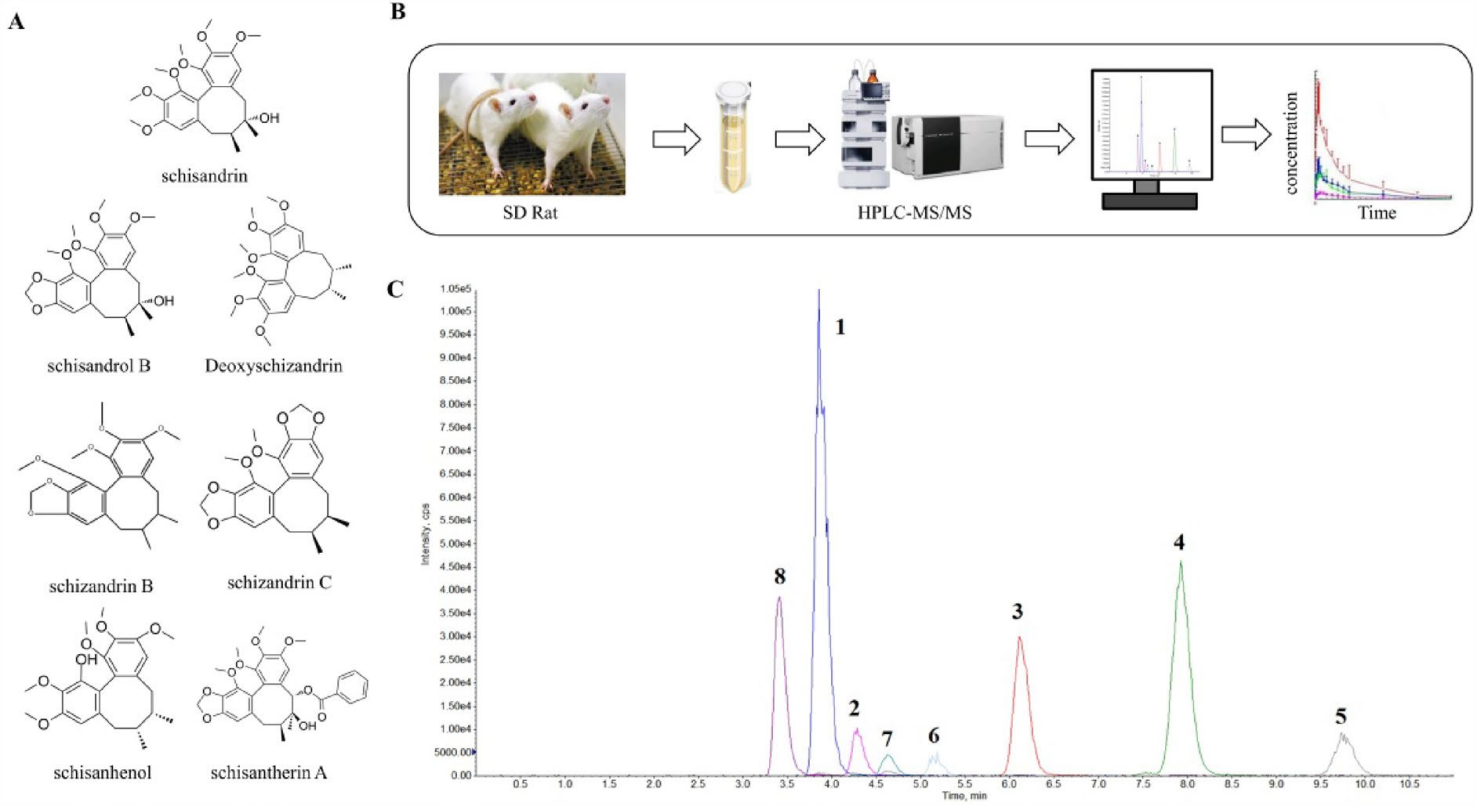
SCH is the quality marker of SC. **A** Chemical structural formulas of seven main lignans in SCE; **B** Determination of seven main lignans in rat plasma by HPLC–MS/MS method; **C** Chromatograms of seven lignans after oral administration of SC extract for 2 h. 1.SCH; 2.*Schisandrol B*; 3.*Schisandrin A*; 4.*Schisandrin B*; 5. *Schisandrin C*; 6. *Schisanhenol*; 7. *Schisantherin A*; and 8. IS

All seven lignans were detected within 15 min of rats received 0.95 g/kg SCE, indicating their rapid absorption into the bloodstream in *vivo* (Fig. 1). Table 2 displays the analysis results of the main pharmacokinetic (PK) parameters of the seven lignans. Among them, the C_max_ and AUC_0-∞_ of SCH were significantly higher than those of other lignans, which were (1078.54 ± 179.30) ng/mL and (5775.05 ± 1457.02) ng.h/mL, respectively. Therefore, it can be speculated that SCH has a potential UC impact in the presence of other lignan components with anti-ulcerative colitis effects and similar chemical structures Fig. 1A.

**Table 2:** The main pharmacokinetic parameters of seven lignan components after SCE gavage in rats (x ± s, n = 6)

Parameters	Schisandrin	Schisandrol B	Deoxyschizandrin	Schizandrin B	Schizandrin C	Schisanhenol	Schisantherin A
T_1/2_(h)	1.68 ± 0.26	3.30 ± 0.55	4.19 ± 0.50	4.49 ± 0.02	7.15 ± 4.48	6.84 ± 2.62	5.18 ± 3.24
T_max_(h)	2.00 ± 0.00	2.00 ± 0.00	2.67±1.16	2.00 ± 0.00	2.00 ± 0.00	4.00 ± 0.00	2.67 ± 1.16
C_max_(ng/ml)	1078.54 ± 179.30	182.43 ± 19.98	136.74 ± 45.83	183.43 ± 66.96	24.65 ± 9.03	85.31 ± 8.10	75.81 ± 34.20
MRT_0-∞_(h)	4.54 ± 0.70	5.75 ± 0.46	6.23 ± 0.77	9.03 ± 0.34	9.94 ± 4.56	11.09 ± 2.83	8.24 ± 1.24
CL(L/h/kg)	4.62 ± 1.22	7.43 ± 2.02	9.68 ± 3.25	5.41 ± 1.47	10.17 ± 3.73	1.35 ± 0.18	1.25 ± 0.26
V(L/kg)	11.14 ± 3.19	1419.21 ± 412.19	59.85 ± 25.71	35.04 ± 9.69	90.87 ± 28.97	12.93 ± 3.67	8.71 ± 3.71
AUC_0-t_(ng.h/ml)	5774.32 ± 1456.47	1419.21 ± 412.19	946.23 ± 379.15	1615.77 ± 439.42	195.09 ± 87.05	1115.96 ± 160.10	672.45 ± 139.56
AUC_0-∞_(ng.h/ml)	5775.05 ± 1457.02	1431.21 ± 410.25	960.14 ± 380.61	1627.57 ± 440.60	224.67 ± 129.91	1160.33 ± 205.58	709.09 ± 189.60

### The effect of SCH on UC mice

**Fig. 2:**
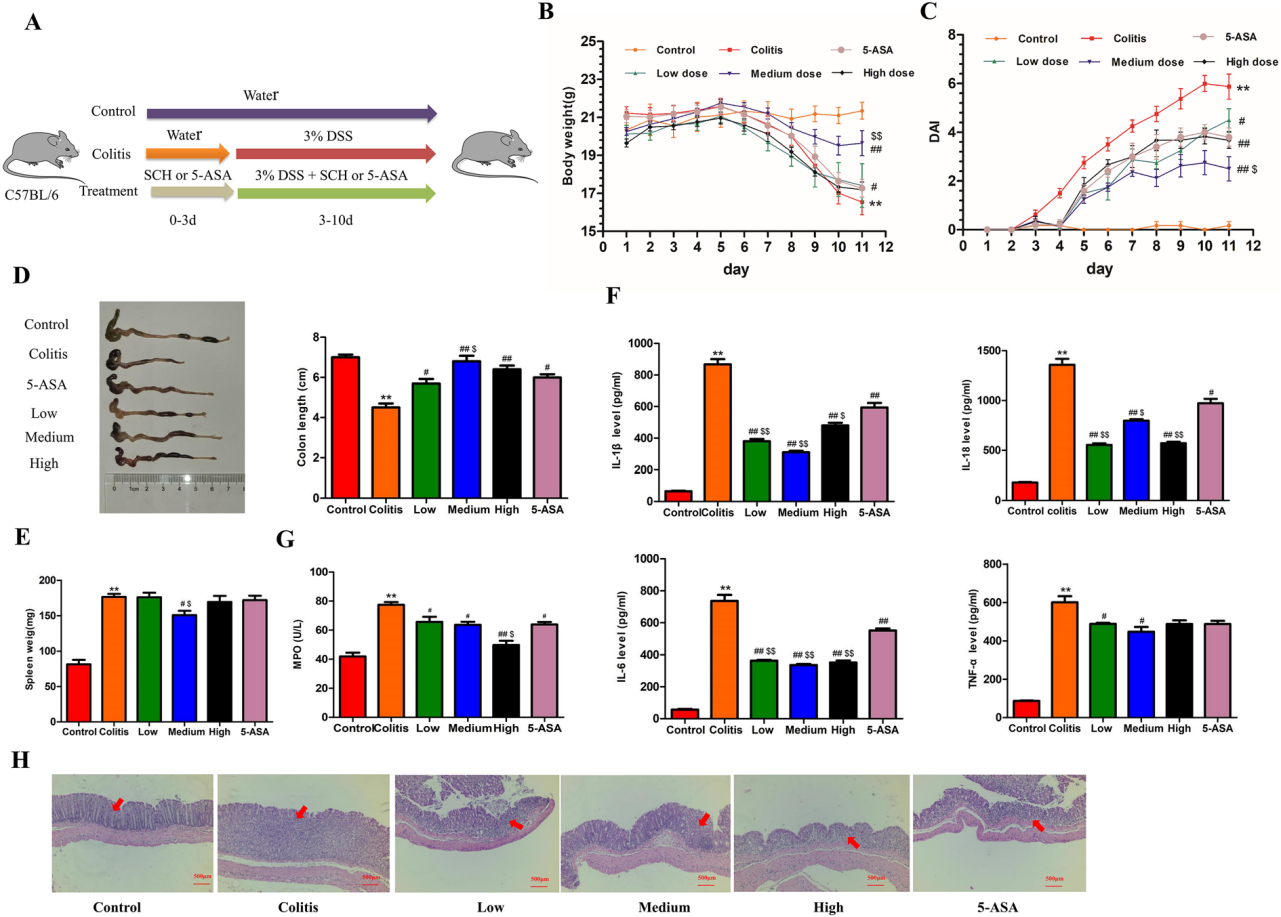
SCH effects on UC mice. **A** Experiment design; **B** Daily changes of body weight in different groups; **C** Daily changes of disease activity index (DAI) in different groups; **D** Colon length; **E** Ratio of spleen to body weight; **F** Serum levels of TNF-α, IL-6, IL-1β and IL-18; **G** Serum levels of MPO; **H** Representative pictures of **H** and **E**-stained colon tissue. Scale bar = 500 μm. Data represent the mean ± SEM (n = 8). **p < 0.01 vs Control group, ^#^*p* < 0.05, ^##^*p* < 0.01 vs Colitis group,^$^*p* < 0.05, ^$$^*p* < 0.01 vs 5-ASA group

By comparing the enteritis phenotypes of mice in each group, it can be observed that the mice in the administration group exhibited slower weight loss compared to the model group. The symptoms of bloody stool were relieved, and the DAI value was lowered. In addition, colon length and quality were significantly better than those in the model group (Fig. 2B–D). Spleen weight is an important indicator of systemic inflammation. Based on the spleen measurement results of each group, it can be concluded that the administration group can significantly improve the splenomegaly of mice with colitis (Fig. 2E).

Compared with the model group, the levels of TNF-α, IL-6, IL-1β, IL-18, and MPO in the serum of therats (mice) in the administration group were significantly reduced (*P* < 0.01) (Fig. 2F, G), indicating a reduction in MPO activity and in vivo inflammation levels. According to H&E staining, the colon tissue in the control group appeared intact with clear structural stratification, showing no signs of damage or inflammatory reaction. In contrast, mice in the model group exhibited extensive mucosal ulcers, infiltration of inflammatory cells, destruction of surface epithelium, and a reduced number of goblet cells. These conditions were significantly improved by SCH treatment (Fig. 2H). The degree of damage to the colon tissue in the administration group was significantly reduced, and the colon tissue structure in the medium dose group appeared clear with a reduction in the infiltration of inflammatory cells in the lamina propria. Moreover, the number of goblet cells was relatively increased, indicating a restoration of tissue morphology.

Although the positive drug 5-ASA alleviated the symptoms of colitis in mice in the above-mentioned aspects, it still paled in comparison to SCH, particularly at medium doses. Therefore, it can be concluded that SCH plays an important role in the phenotypic restoration of DSS-induced colitis and the reduction of inflammation in vivo.

**Fig. 3:**
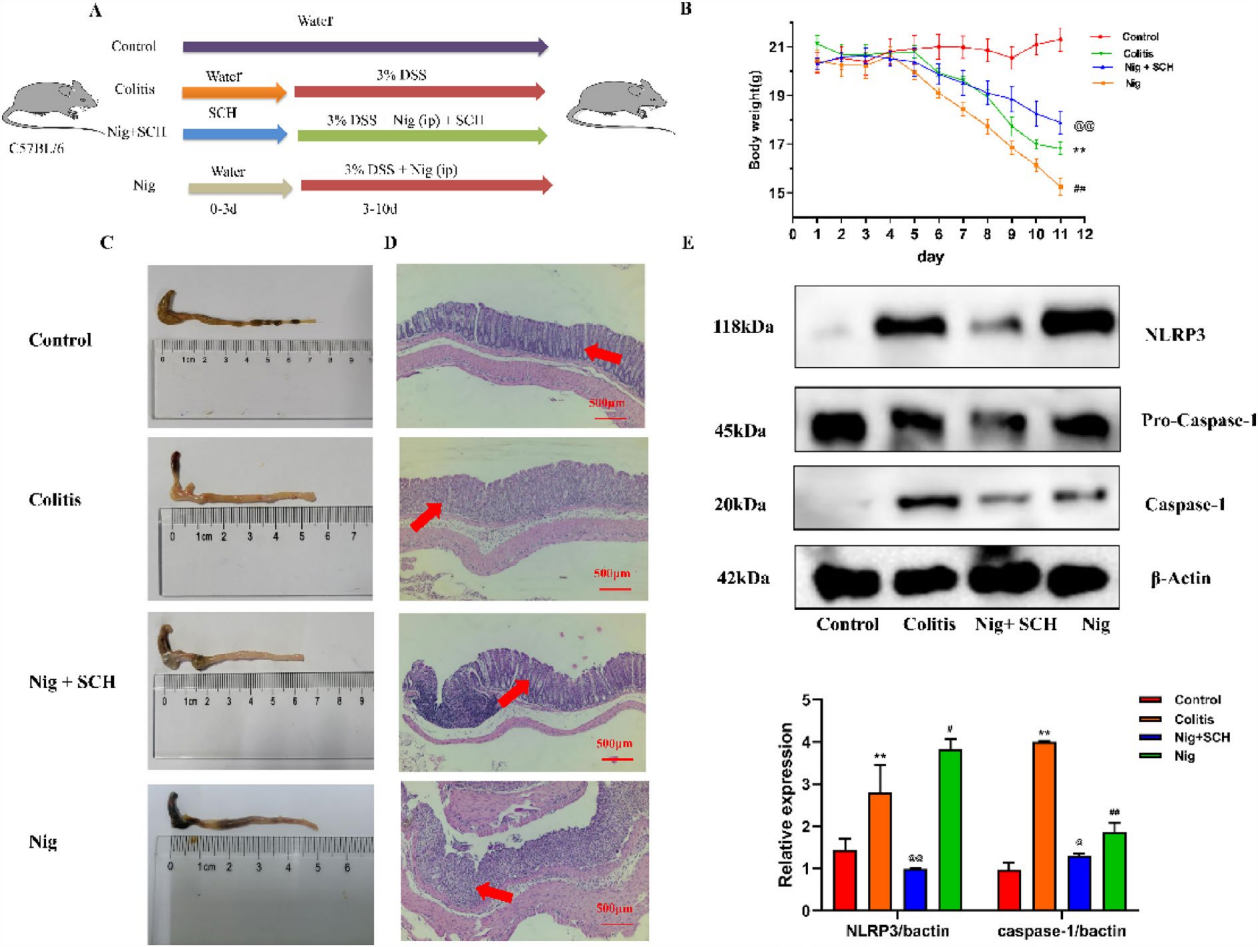
SCH suppressed NLRP3 inflammasone in vivo model of colitis. **A** Experiment design; **B** Daily changes of body weight in different groups; **C** Colon length; **D** The protein expression of NLRP3 and caspase-1 in mice colon tissue. Data represent the mean ± SEM (n = 6). **E** Western blot results **p < 0.01 vs Control group, #*p* < 0.05 vs Colitis group; ##*p* < 0.01 vs Colitis group. @*p* < 0.05 vs SCH + Nig group; @@*p* < 0.01 vs SCH + Nig group

The intraperitoneal injection of Nig revealed that, compared with the Nig group, SCH + Nig significantly slowed down the weight loss trend of mice, restored colon length, and reduced inflammatory cell infiltration (Fig. 3A–D). Western blot was used to detect the protein expression levels of NLRP3 inflammasome and activation marker factor Caspase-1, and the results are shown in Fig. 3E. The expression levels of NLRP3 and Caspase-1 in the model group were significantly higher than those in the control group. The expression levels of NLRP3 and Caspase-1 in the Nig group were significantly higher than those in the model group. However, in the SCH + Nig group, NLRP3 and Caspase-1 expression were significantly decreased, and the expression of Caspase-1 was comparable to that of the control group. These results indicate that in a DSS-induced mice model of UC, SCH effectively inhibits the activation of the NLRP3 inflammasome.

### Effect of SCH on mucus layer and goblet cells in colon tissue of UC mice

**Fig. 4:**
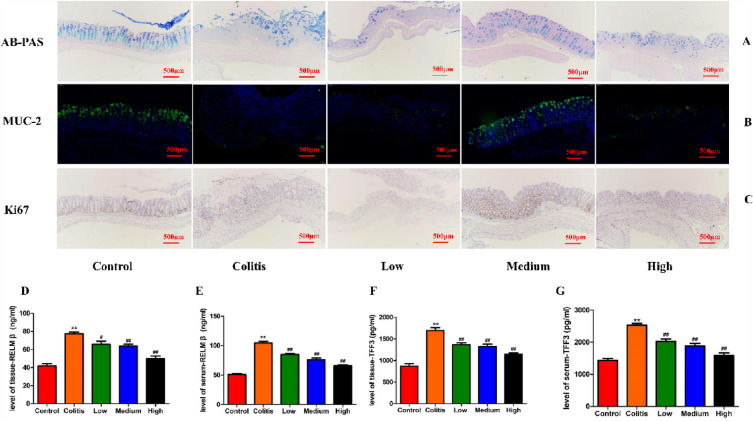
The protective effect of SCH on goblet cells. **A** AB-PAS staining; **B** Immunofluorescence staining for MUC-2 in colon tissue sections of experimental mice, green section; **C** Expression of Ki67 in colon tissue (immunohistochemistry × 500), red section; **D**–**E** RELM levels in colon tissue and serum samples; **F**–**G** TFF3 levels in colon tissue and serum samples. Data were expressed as mean ± SEM (n = 8).**p < 0.01 vs Control group, ^#^*p* < 0.05, ^##^*p* < 0.01 vs Colitis group

The severity of colitis is associated with damage to the epithelial barrier [26]. MUC-2, secreted by goblet cells, plays a crucial role in maintaining the integrity of the epithelial barrier. Alterations in the quality and quantity of MUC-2 can result in increased permeability, allowing pro-inflammatory substances from the intestinal lumen, such as antigens and endotoxins, to enter the mucosal lamina propria. This process ultimately triggers an immune response and leads to the development of intestinal inflammation. Figure 4A, B depicts the results of AB-PAS staining and immunofluorescence staining of colon tissue. Compared to DSS-induced colitis mice, the medium dose and high dose groups exhibited restoration in the thickness of the mucus layer and the number of goblet cells (Fig. 4A). The intestinal lumen of the control group exhibited abundant mucus, extending from the crypts to the mucosa, accompanied by consistently high expression of MUC-2 protein. The level of MUC-2 protein in the medium dose group exhibited a significant increase, and the site and intensity of MUC-2 protein expression gradually approached that of the control group. According to immunohistochemistry, the medium dose group showed effective protection of intestinal epithelium [27] proliferation compared to the colitis group (Fig. 4C). The results of RELMβ and TFF3 content assay are depicted in Fig. 4D–G. SCH treatment significantly reduces the levels of RELMβ and TFF3 in tissues and serum. In conclusion, a dose of 40 mg/kg SCH exhibits a favorable protective effect on goblet cells. Specifically, it preserves the mucus layer, promotes goblet cell proliferation, and mitigates intestinal barrier damage in DSS-induced colitis.

### DEGs search and bioinformatics analysis

**Fig. 5:**
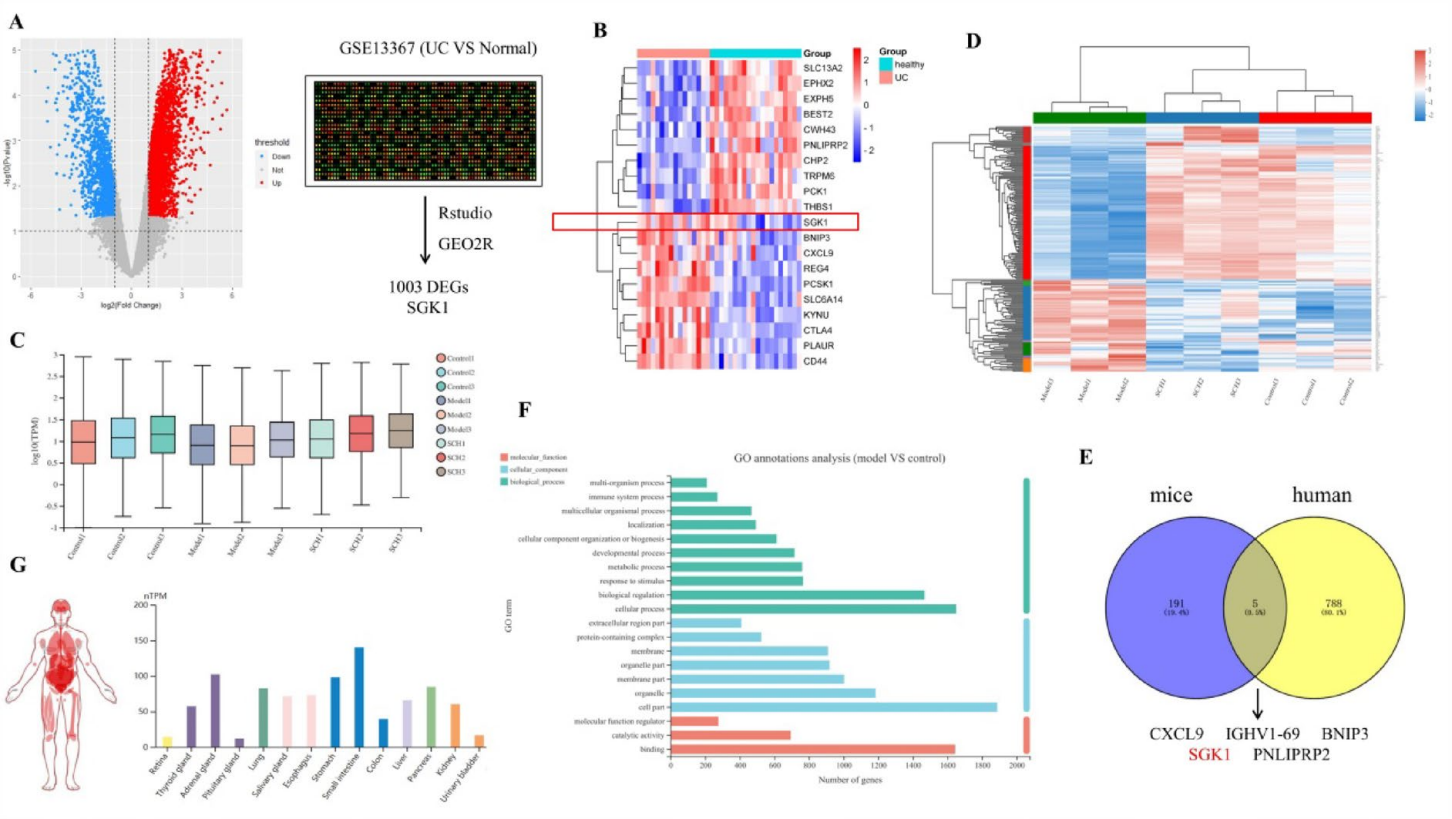
Differential expression of SGK1 in UC patients and mice. **A** Volcano plot of DEGs distribution in GSE13367; **B** Heat map of some differentially expressed genes in GSE13367; **C** Expression of transcriptome data in colon tissue; **D** Heat map of differentially expressed genes in the transcriptome data of three groups; **E** The intersection of human differentiated genes and mice DEGs; **F** GO analysis of DEGs; **g** SGK1 expression in various tissues from Human Protein Atlas (HPA) database

GSE13367 contains a total of 28 UC and healthy human samples. The volcano map and heat map of the dataset are shown in Fig. 5A, B, which represent 1003 differential genes. The transcriptomes of 9 samples generated a total of 67.39 Gb of clean data, with each sample yielding clean data exceeding 6.75 Gb. The percentage of Q30 bases in the data exceeded 94.35%. A total of 197 genes showed no significant difference from the control group but exhibited significant differences compared to the model group (Fig. 5C, D). Analysis of the differential gene expression in the UC transcriptomes of humans and mice revealed five common genes, namely CXCL9, IGHV1–69, BNIP3, SGK1, and PNLIPRP2 (Fig. 5E). Through analysis using the HPA database and GO analysis (Fig. 5F, G), it was observed that SGK1 exhibits high expression in intestinal tissue.

**Fig. 6:**
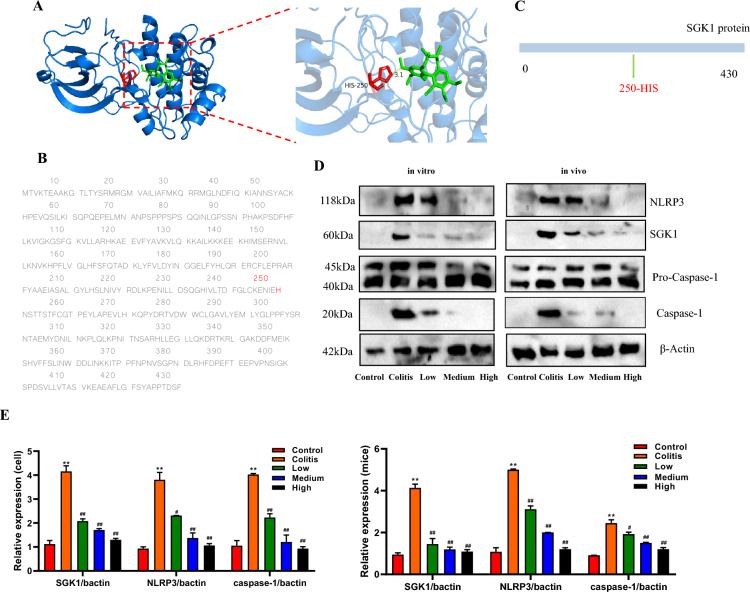
Molecular docking and validation of related protein results. **A** Protein spatial structure and drug structure; SCH may regulated SGK1 protein at 250 site; **B** Amino acid sequence for SGK1 protein; **C** Schematic diagram of protein mutation; **D** SCH suppressed NLRP3 inflammasone in vivo and *vitro* model of colitis; **E** Relative expression

Furthermore, studies have demonstrated that inhibiting SGK1 negatively regulates NLRP3 inflammasome activation [28, 29], protects cardiac function [30], enhances glial activity, and prevents glial cell senescence and mitochondrial damage [31]. The binding energy between the receptor and the ligand is < −5.0 kcal/mol, indicating a strong binding activity between them, as shown in Fig. 6A. The predicted binding site is most likely located at position 250 (Fig. 6B). Therefore, SGK1 was selected for further experiments.

Based on the in *vitro* and in *vivo* results (Fig. 6D, E), it was observed that SCH could suppress SGK1 expression and NLRP3 inflammasome activation, while not affecting the expression of pro-caspase-1 precursor protein. Consequently, this inhibition can effectively reduce caspase-1 levels, leading to the inhibition of IL-1β secretion and IL-18 secretion.

### Regulation of SCH on intestinal microbiota imbalance in colitis mice

#### Evaluation of colon content sequencing results

**Fig. 7:**
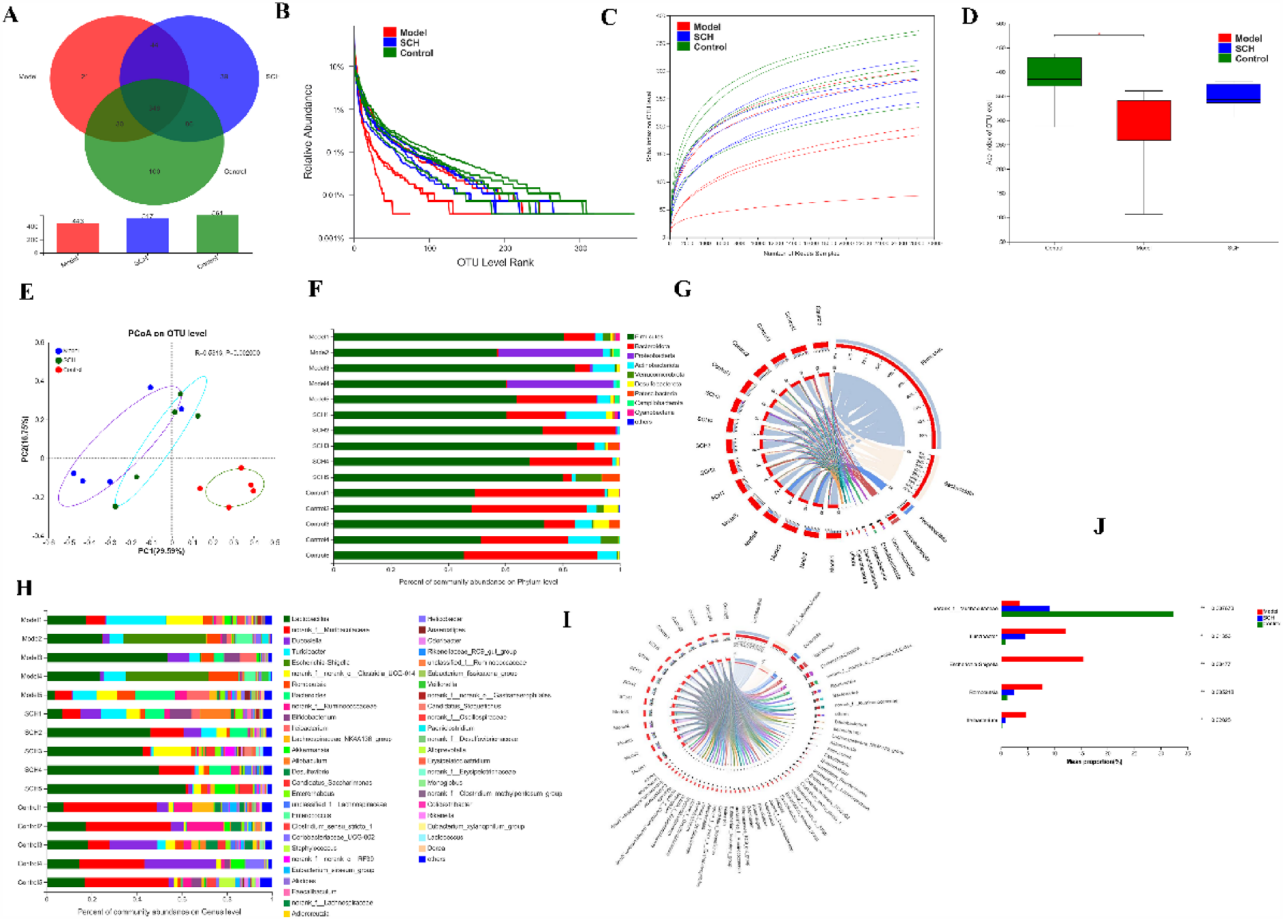
SCH restores gut microbiota balance in DSS-induced colitis mice. **A **The result of OTU analysis; the number represents the number of OTU; **B** Relative abundance of gut microbiota; **C** Sample dilution curve; **D** ACE index box chart; **E**Multiple sample PCoA analysis based on Bray–Curtis similarity, where each point represents a sample, and the samples in same group is represented by the same color (*p* = 0.002); **F**–**G** Relative abundance of gut microbiota at the phylum level; **H**–**I** Relative abundance of gut microbiota at the genus level; **J** Statistical analysis of significant difference test between groups. **p < 0.01, *p < 0.05

In illumina high-throughput sequencing, a total of 668 OTUs and 819,285 raw reads were obtained. After paired-end quality control splicing, a total of 719,857 Clean Reads were obtained, with an average sequence length of 421. According to the detection results of OTU species in each group, there were 387 OTUs in the control group, model group, and SCH group, as well as 100, 21, and 39 unique OTU species in each group, respectively (Fig. 7A). Figure 7B, C depicts the correlation curve between sequencing amount and OTUs. After the sequencing amount reaches a certain value, the sample curve tends to be flatten, indicating that the amount of sequencing data in this experiment meets the analysis requirements.

#### Microbial diversity analysis of colon contents

Alpha diversity analysis can reflect the abundance of flora through the ACE index. A higher ACE index indicates a higher abundance of flora and greater diversity. As shown in Fig. 7D, there was a significant difference in the ACE index between the control group and the model group (*p* < 0.05). To assess the differences in GM composition among the samples in each group, the Principal Coordinates Analysis (PCoA) method was employed to analyze the β-diversity of the GM in mice from each group (Fig. 7E). The distance between the samples of the model group and the samples of the control group was relatively large, indicating a significant difference in the composition of GM. Most of the samples in the SCH group were closer to the control group than the model group, indicating that the SCH treatment induced changes the GM structure of the mice to a certain extent and gradually approached the state of normal flora.

#### Annotation and variance analysis of gut flora species

The GM of each group was analyzed at the phylum and genus levels to further evaluate the effect of SCH on the GM of UC mice. As shown in Fig. 7F, G, *Firmicutes* and *Bacteroidet*es were most abundant phyla in the gut of mice of mice in each group. The proportion of *Bacteroidetes* in the model group was significantly lower than that in the control group, which is consistent with clinical IBD patients and animal models. After SCH treatment, the abundances of *Firmicutes* and *Bacteroidetes* were similar to those of the control group, suggesting ting that SCH intervention can regulate the imbalance of GM in colitis mice. More than 40 genera were identified at the genus level, including *Lactobacillus*, *Norank_f_Muribaculaceae, Dubosiella, Turicibacter,* and *Escherichia-Shigella*. The GM exhibit varying levels of abundance and dominance. Specifically, the abundance of *Lactobacillus* in the model group was significantly lower compared to the control group. However, after SCH intervention, the proportion of *Lactobacillus* in the SCH group significantly increased (Fig. 7H, I). This indicates that SCH intervention effectively enhances the proportion of probiotics and facilitates the recovery of the GM. Furthermore, the levels of *Turicibacter, Romboutsia, and Escherichia-Shigella* were significantly higher in the DSS-induced mice colitis model compared to the control group. After SCH treatment, the abundance of *Turicibacter* and *Escherichia-Shigella* decreased significantly and returned to normal levels. Thus, SCH intervention modulate the GM structure and biodiversity in colitis mice by selectively promoting the growth of probiotic *Lactobacillus* and *Norank_f_Muribaculaceae* and inhibiting the growth of *Turicibacter* and *Escherichia-Shigella*, which partially reversed the GM imbalance and restored intestinal homeostasis in colitis mice.

### Regulation of SCH on BAs metabolism disorder in colitis mice

**Fig. 8:**
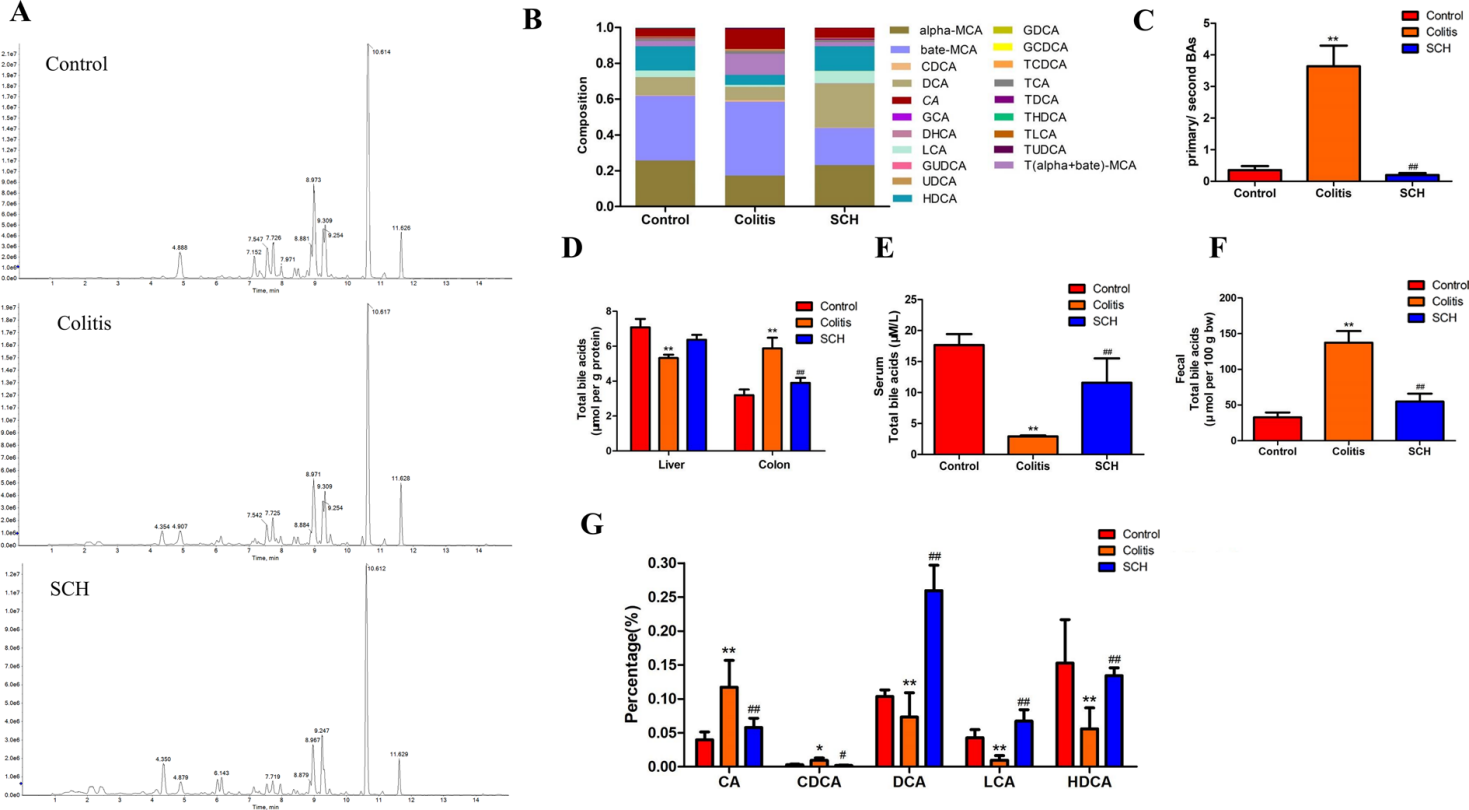
SCH promoted the conversion of primary bile acids to secondary bile acids and significantly increased the proportion of HDCA. **A** HPLC–MS/MS chromatograms of 20 bile acids in control, model, and SCH groups; **B**Composition of bile acid pool in intestinal contents; **C** Ratio of primary/second BAs in intestinal contents; **D** Changes of total bile acid content in colon and liver tissue; **E** Change of total bile acid content in serum; **F** Change of total bile acid content in intestinal contents; **G** Relative abundance of the significantly-changed BAs from different groups

BAs molecules were precisely quantified in the intestinal contents of 20 different types of mice, including Cholic acid (CA), deoxycholic acid (DCA), Lithocholic acid (LCA), ursodeoxycholic acid (UDCA), Chenodeoxycholic acid (CDCA), and others (Fig. 8A, B). Among them, the ratio of primary BAs/secondary BAs was increased in mice under UC state, indicating that GM dysbiosis resulted in weakened metabolic function. In this case, primary BAs could not be effectively converted into secondary BAs (Fig. 8C). After SCH treatment, the ratio showed a downward trend. By measuring the total BAs content in colon tissue, liver tissue, serum, and intestinal contents, it was found (Fig. 8D, E and F) that the total BAs level were lower in serum and higher in colon and intestinal contents in mice with colitis. After SCH treatment, it changed and tended towards the control group, which was consistent with previous findings [32]. The results showed that SCH could restore normal liver and serum BAs levels to a certain extent, which may be related to the promotion of hepatic-intestinal circulation. Through further statistical analysis of the proportion of bile acid molecules in each group, it can be concluded that SCH can significantly increase the proportion of LCA, DCA, and Hyodeoxycholic acid (HDCA) (Fig. 8G), which was correlated with the increase in the abundance of beneficial bacteria such as *Lactobacillus* [33]. Related studies have shown that DCA and LCA can bind to the bile acid receptor TGR5 or FXR and exert immune effects, such as maintaining intestinal barrier homeostasis and inhibiting the release of epithelial cytokines [34, 35].

When SCH was used to treat mice with colitis, it increased the ratio of DCA, LCA, and HDCA in the intestine. It had a regulatory effect on BAs disturbance and can partially restore the BAs content in the liver and serum.

## Discussion

Due to its high safety factor and few toxic effects, TCM offers distinct advantages in the prevention and treatment of chronic diseases. Numerous isolated monomers from TCM have shown potential benefits in various of inflammatory diseases, including UC [36, 37]. In recent years, there has been a lot of interest in finding monomers that have a significant curative effect while being less toxic and having fewer side effects [38–40]. PK experiments in this study demonstrated that SCH is a quality marker of SC. Building upon existing research and literature reports from our research group [41], we hypothesized that SCH exhibits potential pharmacological effects in clinical cases of UC. To further explore the mechanism of SCH against UC, we conducted a series of investigations and verifications encompassing physical, chemical, and biological barriers, providing valuable insights for future research.

**Fig. 9:**
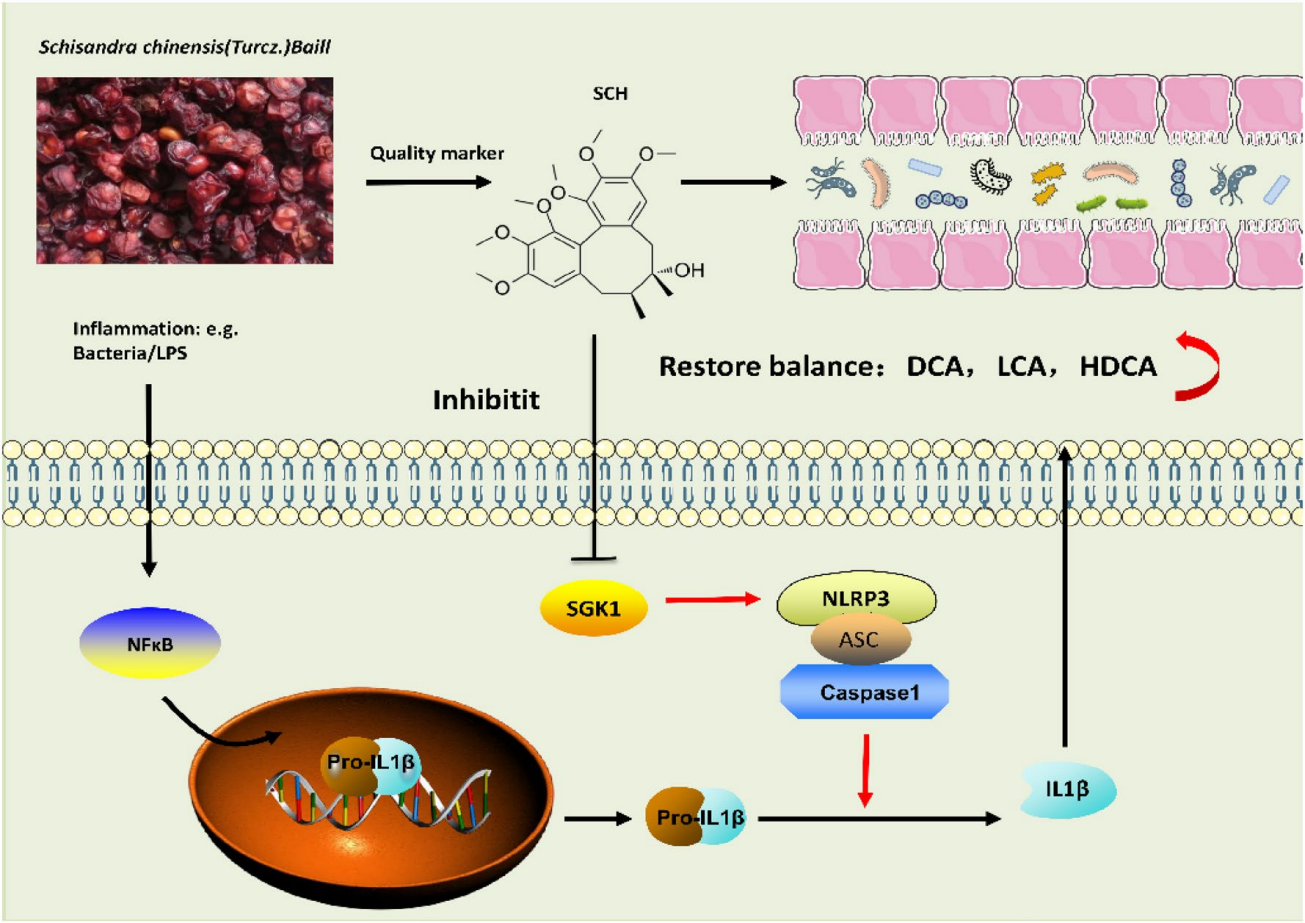
SCH Protects against UC by Inhibiting the SGK1/NLRP3 Signaling Pathway and reshaping Gut Microbiota (GM) in Mice (1.SCH as a quality marker of SC; 2.Increase the proportion of beneficial strains; 3.Restore the balance of bile acids in mice with UC)

In this study (Fig. 9), UC was induced in mice using DSS, and for the first time, the therapeutic effect of SCH on UC mice was confirmed [9, 42]. The mice exhibited symptoms such as decreased appetite, weight loss, fecal blood sample, and colon shortening after DSS induction, as well as corresponding histopathological changes such as neutrophil infiltration, disappearance of goblet cells, and mucosal damage.

In PK experiments, Cao et al. discovered that SCH promotes rapid absorption and elimination in vivo [43] and demonstrated that the main metabolic enzyme of SCH with low bioavailability is CYP3A [44, 45]. Despite this, SCH still has a significant therapeutic effect on UC, indicating that SCH possesses the potential to be developed for clinical practice.

The NLRP3 inflammasome is a cytoplasmic multi-protein complex composed of NLRP3, ASC, and pro-caspase 1. This experiment demonstrates for the first time that SCH inhibits Nig and ATP-induced NLRP3 inflammasome activation and pro-caspase-1 cleavage, thereby inhibiting IL-1β secretion. Intriguingly, SGK1 inhibition was suggested to suppress NLRP3 inflammasome activation. We discovered that SCH could significantly cuts down SGK1, which has been linked to UC [46, 47], content in colon tissue. In UC patients, the expression of SGK1 is increased in peripheral blood mononuclear cells, particularly in CD4 + cells. Hence, SCH may exert its inhibitory effects on NLRP3 inflammasome activation by negatively regulating SGK1. However, compared to existing studies [48], the following issues remain unclear in the present study: how SCH affects immune cells in colonic tissue, and how NLRP3 inflammatory vesicles and GM interact with each other [49]. We hope that such questions could be explored further on follow-up experiments.

Active ingredients and compounds derived from Traditional Chinese Medicine (TCM) have shown effective alleviation of UC symptoms, and the regulation of GM imbalance represents an important approach in TCM-based UC treatment [50, 51]. Likewise, BAs play important roles in the regulation of intestinal immunity and inflammation [52].

Alterations in GM structure during UC lead to changes in the composition ratio of BAs, thereby disrupting the intestinal immune balance and compromising the biological barriers. BAs possess the ability to directly influence the overall metabolism of intestinal bacteria and reshape the GM by altering the composition and structure of the flora.

There is no doubt regarding the interaction between GM and BAs. GM plays a role in converting primary BAs to free BAs through bile salt hydrolase, and these free BAs undergo dehydroxylation to form secondary BAs. However, the accumulation of secondary BAs may have detrimental effects on the structure and function of the colonic epithelium, including the induction of oxidative DNA damage and inflammation. There is also evidence supporting the bidirectional interaction between NLRP3 inflammasome and GM, which plays a role in maintaining intestinal homeostasis [53, 54]. The interaction between GM and NLRP3 inflammasome is crucial for the dynamic balance of the gut. For instance, NLRP3 activation in the gut enhances the release of IL-1β, which subsequently increases local antimicrobial peptide levels, resulting in a shift in GM towards an anti-inflammatory phenotype [55].

Metabolites produced by GM, such as short chain fatty acids (SCFAs), contribute to a shift in GM towards an anti-inflammatory phenotype. SCFAs promote NLRP3 inflammasome activation, which in turn regulates the expression of tight junction proteins, thereby maintaining the integrity of the intestinal epithelial barrier. Therefore, it is essential to investigate the interplay among GM, BAs, and NLRP3 inflammasome in the context of colitis.

We investigated the composition of GM and BAs and observed that SCH played a role in rebalancing the GM and significantly increasing the proportion of secondary BAs. This effect may be associated with the abundance of *Lactic acid bacteria*. Additionally, SCH significantly increased the proportion of LCA, DCA, and HDCA. LCA and DCA levels have been reported to be significantly lower in UC patients compared to controls [56]. HDCA, on the other hand, has demonstrated a potent anti-inflammatory effect in DSS-induced colitis by downregulating inflammatory cytokines [57]. Supplementation of HDCA to mice has also shown significant alleviation of the systemic inflammatory response [58]. However, it should be noted that HDCA has also been shown to inhibit intestinal epithelial cell proliferation through the FXR-PI3K/AKT pathway [59]. It is possible that HDCA has a greater impact on inflammatory cytokines, but further investigation is needed to confirm this.

## Conclusion

SCH exhibited remarkable therapeutic effects in DSS-induced UC mice, leading to significant improvements in UC symptoms, as well as the pathological and histological condition of the colon. These effects may be attributed to the inhibition of SGK1, resulting in the negative regulation of NLRP3 inflammasome activation, modulation of the release of inflammation-related factors, and restoration of GM homeostasis.

## Data Availability

The datasets used and/or analyzed during the current study are available from the corresponding author on reasonable request.

## Abbreviations

BAs: Bile acids
CMC‑Na: Sodium carboxymethylcellulose
DSS: Dextran sulfate sodium
DAI: Disease activity index
DEGs: Differentially expressed genes
ELISA: Enzyme‑linked immunosorbent assay
5‑ASA: 5‑Aminosalicylic acid
GM: Gut microbiota
GEO: Gene expression omnibus
H&E: Haematoxylin and eosin
HPA: Human Protein Atlas
HPLC–MS: High Performance Liquid Chromatography-Mass Spectrometry
IL-6: Interleukin 6
IL-18: Interleukin 18
IBD: Inflammatory bowel disease
LPS: Lipopolysaccharide
LEfSe: Linear discriminant analysis effect size
MPO: Myeloperoxidase
NCBI: National Center for Biotechnology Information
OTUs: Operational taxonomic units
PBS: Phosphate buffered saline
SC: Schisandra chinensis (Turcz.) Baill
SGK1: Serum/Glucocorticoid Regulated Kinase 1
SCE: *Schisandra chinensis (Turcz.)* Baill. Extract
SCH: Schisandrin
TNF-α: Tumor necrosis factor-α
TCM: Traditional Chinese Medicine
UC: Ulcerative colitis
WB: Western blotting
